# Are the Temporal Trends of Stomach Cancer Mortality in Brazil Similar to the Low, Middle, and High-Income Countries?

**DOI:** 10.3389/fpubh.2021.677012

**Published:** 2021-06-29

**Authors:** Samantha Hasegawa Farias, Wilson Leite Maia Neto, Katia Pereira Tomaz, Francisco Winter dos Santos Figueiredo, Fernando Adami

**Affiliations:** Epidemiology and Data Analysis Laboratory, Centro Universitário Saúde ABC, Santo André, Brazil

**Keywords:** epidemiology, stomach neoplasms, socioeconomic status, mortality, social inequalities

## Introduction

Stomach cancer is the fourth most common malignant tumor in the world, and although numbers have fallen in recent years, mortality from this cause is still high (1–3). In Brazil, some studies have shown a reduction in mortality from stomach cancer since the 1980s (4, 5), which can be attributed to improved eating habits, food preservation, and treatment of Helicobacter pylori infection (6, 7).

In addition, there were significant advances related to socioeconomic development and the reduction of inequalities and socioeconomic inequities, which improved the population's access to health care and reduced the morbidity and mortality of diseases such as breast cancer (8–10).

Brazil is a middle-income country characterized by great internal heterogeneity (11, 12). It is notorious that poverty in Brazil has a location (13) and, in terms of disparities, the country has a very striking feature that is the regional inequalities, where the north and northeast regions have the worst indicators. The central region has intermediate rates, and the south and southeast are the best conditions, regardless of the socioeconomic indicator being evaluated (14). These social inequalities in the country still today directly reflect on health inequality, explaining the unfavorable scenarios for the north and northeast, and a very evident polarization in relation to the south and southeast (15).

The country presents regions with different socioeconomic characteristics, which impacts health services, lifestyle, and socio-cultural aspects. In other words, there are developed regions with high technology for cancer-oriented health services and underdeveloped regions that cannot properly treat and diagnose its citizens (16).

Thus, considering that Brazil is a country with territorial extension of continental characteristics and high socioeconomic plurality, and that the mortality due to stomach cancer is related to the socioeconomic status of the site, what level of development does the behavior of stomach cancer mortality in Brazil follow?

Thus, the aim of this study was to describe the temporal trend of stomach cancer mortality in Brazil from 1990 to 2016, analyzing its behavior in relation to low, middle, and high income countries.

## Methods

### Study Design

Secondary data analysis performed based on data from 1990 to 2016 obtained from the Global Burden of Disease (GBD).

### Data Source

The Global Burden of Disease database is coordinated by the Institute of Health Metrics and Evaluation (IHME) of the University of Washington and maintained through a partnership with researchers from 124 countries, with the objective of estimating the global burden of more than 300 diseases and injuries (17).

This database provides information from various sources based on official documents such as censuses, administrative databases, scientific publications, hospital and police records, among others. Through this information, there is a joint effort by scientific commissions from various countries to systematically quantify the magnitude of health loss due to diseases, injuries and risk factors by age, sex, and geographic location.

To facilitate the production of estimates and comparability of data, GBD researchers created a measure to classify the socio-demographic development of a locality, the Socio-demographic Index (SDI) (18), based on the average income per person, schooling, and total fertility rate to classify countries as low, medium low, medium, medium high, and high income.

### Study Variables

The studied variables were deaths, age-standardized mortality, and proportional mortality from all cancer causes and proportional to all deaths. Data for Brazil and low, middle, and high income countries were adjusted for age and were expressed as rates (per 100,000 inhabitants). In the present study, only the low, middle, and high income classifications were evaluated, in order to better capture the differences between the analysis groups.

### Statistical Analysis

Descriptive statistics were performed using the statistical program Stata® (StataCorp, L,C) version 11.0 and presented through absolute and relative frequency.

The time trend analysis was performed through the program Joinpoint Regression version 4.6.0 (Statistical Research and Applications Branch, National Cancer Institute, Rockville, EUA) (19). The joinpoint regression is a technique that explores the relationship between two variables by means of segmented linear regression. It determines the magnitude of change in the trend in percentage terms and verifies whether or not this change is statistically significant (20).

The final model chosen was the one with the highest number of points and maintained the statistical significance (*p* < 0.05). From the estimated slope for each straight line (regression coefficient), the Annual Percentage Change (APC) and Average Annual Percentage Change (AAPC) were calculated and its statistical significance was estimated by the Least Squares Method by a generalized linear model and for each straight line segment, with an estimated slope, and their 95% confidence intervals.

### Ethical Aspects

According to Resolution No. 510 of April 7, 2016 of the National Health Council of Brazil, since these are public data and of free access. There is no need for ethical appreciation.

## Results

There were 14,139,731 deaths from stomach cancer in the high, middle, and low income countries between 1990 and 2016, of which 612,818 were in low-income countries, 9,137,851 in middle-income countries and 4,389,062 in high-income countries. In Brazil, there were 449,682 deaths in the same period.

With regard to socioeconomic status, stomach cancer mainly affects middle-income countries. In these countries, ~25 people die from stomach cancer per 100,000 inhabitants, representing 2.3% of all deaths from known causes and 12.3% of deaths from some form of cancer. In Brazil, the burden of stomach cancer appears to be lower than that observed in middle-income countries (15.5 deaths per 100,000 inhabitants, mortality proportional to all deaths of 1.6%, and all cancers of 9.7%) (Table 1).

**Table 1:** Mean mortality rates and age-adjusted mortality rates due to stomach cancer, proportional mortality for all deaths and proportional mortality for all cancers, 1990–2016.

**Place**	**Mortality rate^a^**	**Age-standardized Mortality rate^a^**	**Proportional mortality (%)**	
			**All deaths**	**All cancers**
Brazil	13,10	15,54	1,60	9,74
**Socioeconomic status**				
Low income	11,06	11,12	0,38	8,01
Middle income	17,16	24,70	2,31	12,37
High income	10,78	12,04	1,94	7,44

a *Per 100,000 inhabitants*.

**Figure 1:**
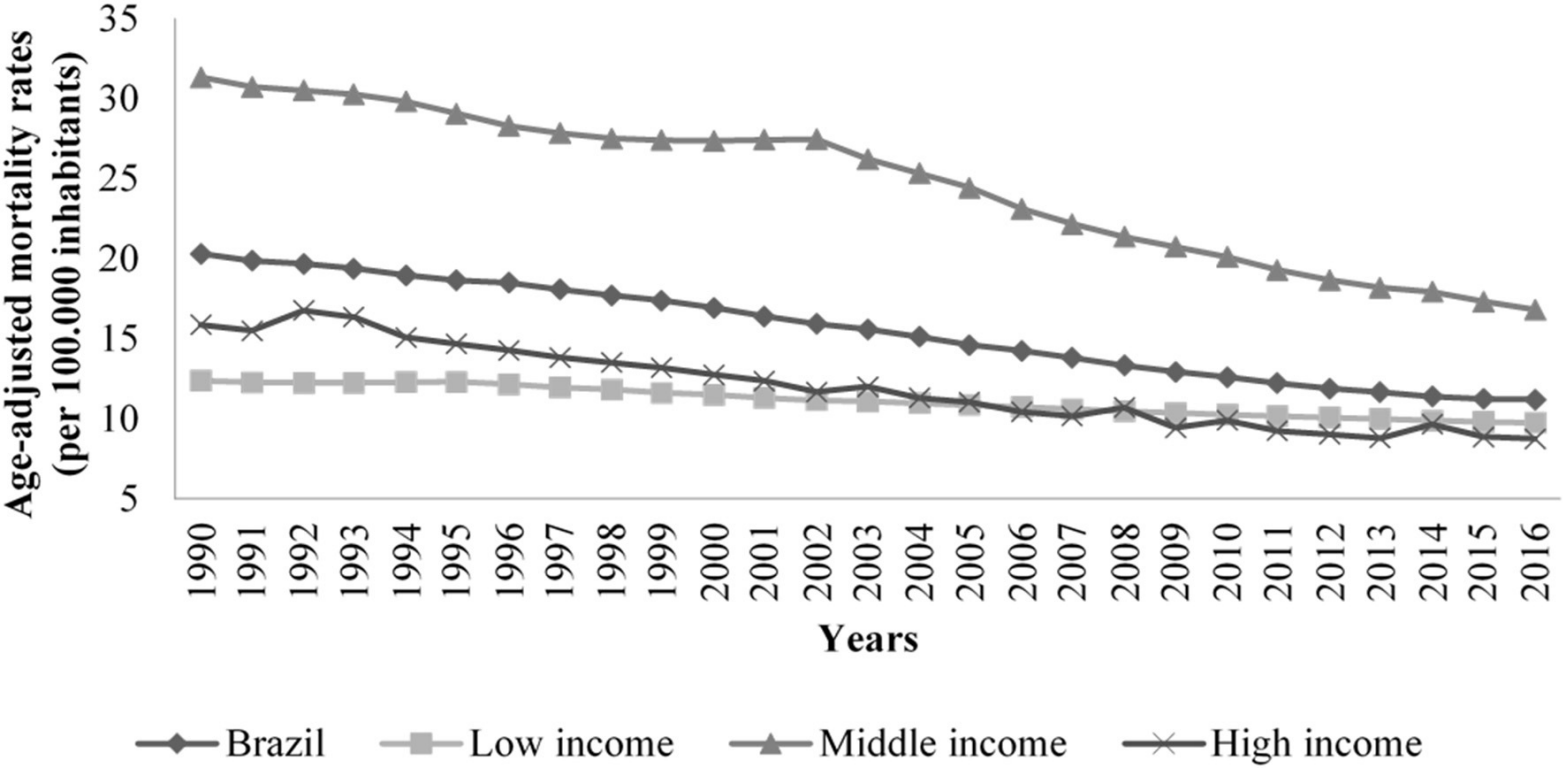
Trends of age adjusted mortality rates related to stomach cancer (per 100,000 inhabitants) in Brazil and low, middle, high income countries, 1990 to 2016.

It was observed that, regardless of the socioeconomic status, there is a decrease in the mortality rates due to stomach cancer in the studied sites. Throughout the study time, the rates decreased more in high income countries, while the middle income countries had greater variability (Figure 1).

In the first period of change corresponding to the years between 1990 and 2003, Brazil presented the annual percentage change (APC) of −1.8 (95% CI −1.9; −1.7), behavior of low and middle-income countries, which presented the same changes in their respective first periods of change. The second period of change observed in Brazil corresponded to the years of 2003–2015 and had APC of −2.8 (95% CI −3.0; −2.7), behavior close to high-income countries, which presented APC of −2.5 (95% CI −2.6; −2.4) (Table 2).

**Table 2:** Estimates of temporal trend of specific mortality rates for stomach cancer according to cut-off points obtained through the joinpoint. 1990–2016.

	**Period**	**AAPC (95%CI)**	**APC(95%CI)**	***p*-value**
Brazil	1990–2003	−2.3 (−2.4; −2.2)	−1.8 (−1.9; −1.7)	<0.001
	2003–2016		−2.8 (−3.0; −2.7)	<0.001
Low income	1990–2004	−1.4 (−1.5; −1.3)	−1.8 (−1.8; −1.7)	<0.001
	2004–2013		−1.2 (−1.3; −1.0)	<0.001
	2013–2016		−0.4 (−1.1; ^_^0.4)^a^	0.3
Middle income	1990–1997	−2.1 (−2.1; −2.0)	−1.8 (−2.0; −1.7)	<0.001
	1997–2004		−0.9 (−1.1; −0.8)	<0.001
	2004–2007		−5.4 (−6.3; −4.6)	<0.001
	2007–2010		−1.8 (−2.7; −1.0)	<0.001
	2010–2013		−3.3 (−4.2; −2.5)	<0.001
	2013–2016		−0.7 (−1.2; −0.3)	<0.001
High income	1990–1995	−2.7 (−2.8; −2.6)	−2.6 (−3.0; −2.2)	<0.001
	1995–2006		−3.0 (−3.1; −2.9)	<0.001
	2006–2016		−2.5 (−2.6; −2.4)	<0.001

*CI, confidence interval; APC, Annual Percent Change; AAPC, Average Annual Percent Change*.

a *The APC is not statistically significant (p > 0.05)*.

When analyzing the average of the annual percentage change, we observed that the low-income countries had the lowest fall with the AAPC of −1.4(95% CI −1.5; −1.3), followed by middle-income −2.1(95% CI −2.1; −2.0) and high income countries −2.7(95% CI −2.8; −2.6). Brazil presented AAPC of −2.3(95% CI −2.4; −2.2).

## Discussion

Between 1990 and 2016, there was a downward trend in age-adjusted mortality from stomach cancer in all socioeconomic statuses studied (low, middle, and high income) and in Brazil, which showed a similar trend to that observed in middle-income countries.

The decrease in mortality in all socioeconomic statuses studied can be explained by the improvement in the population living conditions. Even in poorer countries, there has been improvement in social and economic aspects in recent decades (21).

Despite the improvements, epidemiological studies have found relationships between low socioeconomic status in childhood and the development of stomach cancer in adult life. One of the possibilities would be an early infection by H. pylori bacteria (22, 23). In view of this, it is to be understood that changes in mortality rates in low- and middle-income countries still tend to bear the consequences of this socioeconomic condition over a given time, even if they have already been overcome.

Over time, Brazil presented similar variations to all high-income countries, and in some periods of the series studied, variations were found in both low-income and middle- and high-income countries.

However, the mortality rates presented in Brazil are similar to the rates of middle-income countries and higher than those of some high-income countries (5, 24). This is because despite the high incidence in countries such as Japan, China, and South Korea, the diagnosis of stomach cancer occurs early, which reduces mortality (25).

On the other hand, some factors may explain the higher mortality in Brazil. Cancers of infectious origin, such as the stomach, are common in Latin countries due to economic development, and the Brazilian health system has no guidelines for screening. One of the main aspects that is directly involved with cases of stomach cancer deaths in Brazil is the inequality related to economic, geographic, and socio-cultural issues (5, 26, 27).

Despite the drop in stomach cancer mortality in Brazil, the cases are still high and projections show an increase in the less developed regions of the country (4, 5). This fact underscores the importance of studies that take into account the geographical distribution, especially in countries such as Brazil, characterized by large socioeconomic discrepancies between regions.

It is important to emphasize that the territorial extension of Brazil also has an impact on the difficulty of professional qualification, access to health services and treatment funds, important factors for early detection, clinical management, and patient survival (26, 28).

Another important issue to consider in the current scenario of stomach cancer in the country and that is directly related to territorial extension was the lack of standardization in the diagnosis, staging, and treatment in the study period (26), key factors in achieving good treatment results (29). Only in 2018 did Brazil approve diagnostic and therapeutic guidelines for stomach adenocarcinoma, which is the most common type of gastric cancer, accounting for about 90% of diagnosed cases (30).

Brazil presents a process of demographic and epidemiological transition that occurs differently depending on its Federative Units due to its socioeconomic disparities (31).

The North and Northeast regions present characteristics of low and middle income countries, such as high mortality rates due to infectious diseases (32), worse sanitation conditions (33) and a larger proportion of population residing in rural areas (34).

In contrast, the Midwest, South, and Southeast regions have characteristics of high income countries, such as the increase of chronic diseases such as obesity (35), the increase in life expectancy and, therefore, a more aged population (36).

This scenario shows that Brazil encompasses several factors that may influence the burden of stomach cancer. It is important to identify what local socioeconomic characteristics are related to the disease, which is a crucial starting point for the change of scenery in the country.

The limitations of this study are related to the use of secondary data, in which the researcher does not have control of data quality. However, despite being a constraint, we believe that because it is a database produced by important institutions and the database is used in scientific articles published in high impact journals, the findings support the reliability and validity of this data.

## Conclusion

Over time, Brazil shows a constant decline, with periods of variation similar to the behavior observed in both high and low income countries. Additionally, the findings of this study point to the need to understand the behavior of stomach cancer mortality in the regions and federal states of Brazil, since they present different socioeconomic characteristics.

## Data Availability Statement

Publicly available datasets were analyzed in this study. This data can be found at: http://ghdx.healthdata.org/gbd-results-tool.

## Author Contributions

SF conceived the study, analyzed the data, constructed the results from the data, and wrote the manuscript. WM analyzed the data, constructed the results from the data, and wrote the manuscript. KT collected the data and constructed the results from the data. FF conceived the study, analyzed the data, and reviewed results. FA conceived the study and reviewed results. All authors read and approved the final manuscript.

## Conflict of Interest

The authors declare that the research was conducted in the absence of any commercial or financial relationships that could be construed as a potential conflict of interest.
